# Structural and Ultrastructural Alterations in Human Olfactory Pathways and Possible Associations with Herpesvirus 6 Infection

**DOI:** 10.1371/journal.pone.0170071

**Published:** 2017-01-10

**Authors:** Sandra Skuja, Anete Zieda, Kristine Ravina, Svetlana Chapenko, Silvija Roga, Ojars Teteris, Valerija Groma, Modra Murovska

**Affiliations:** 1 Institute of Anatomy and Anthropology, Riga Stradins University, Riga, Latvia; 2 Department of Neurosurgery, Stanford University School of Medicine, Stanford, California, United States of America; 3 A. Kirchenstein Institute of Microbiology and Virology, Riga Stradins University, Riga, Latvia; 4 Department of Pathology, Riga 1st Hospital, Riga, Latvia; 5 Latvian State Centre for Forensic Medical Examination, Riga, Latvia; Rutgers University, UNITED STATES

## Abstract

Structural and ultrastructural alterations in human olfactory pathways and putative associations with human herpesvirus 6 (HHV-6) infection were studied. The olfactory bulb/tract samples from 20 subjects with an unspecified encephalopathy determined by pathomorphological examination of the brain autopsy, 17 healthy age-matched and 16 younger controls were used. HHV-6 DNA was detected in 60, 29, and 19% of cases in these groups, respectively. In the whole encephalopathy group, significantly more HHV-6 positive neurons and oligodendrocytes were found in the gray matter, whereas, significantly more HHV-6 positive astrocytes, oligodendrocytes, microglia/macrophages and endothelial cells were found in the white matter. Additionally, significantly more HHV-6 positive astrocytes and, in particular, oligodendrocytes were found in the white matter when compared to the gray matter. Furthermore, when only HHV-6 PCR^+^ encephalopathy cases were studied, we observed similar but stronger associations between HHV-6 positive oligodendrocytes and CD68 positive cells in the white matter. Cellular alterations were additionally evidenced by anti-S100 immunostaining, demonstrating a significantly higher number of S100 positive cells in the gray matter of the whole encephalopathy group when compared to the young controls, and in the white matter when compared to both control groups. In spite the decreased S100 expression in the PCR^+^ encephalopathy group when compared to PCR^-^ cases and controls, groups demonstrated significantly higher number of S100 positive cells in the white compared to the gray matter. Ultrastructural changes confirming the damage of myelin included irregularity of membranes and ballooning of paranodal loops. This study shows that among the cellular targets of the nervous system, HHV-6 most severely affects oligodendrocytes and the myelin made by them.

## Introduction

Human herpesvirus 6 (HHV-6) is a DNA-containing, enveloped virus of the *Herpesviridae* family, capable of lifelong persistence in host cells after the primary infection in early childhood [1]. After infecting the host, the viral genome persists in various organs including salivary glands, kidneys and brain and is typically reported in the transplant setting [2,3]. Epidemiological studies demonstrate that at least 80% of the healthy population is seropositive for HHV-6 [4] suggesting establishment of latency in immunocompetent hosts.

HHV-6 virus was first isolated from human peripheral blood lymphocytes [5]. A year later, a new report about the neurotropic potential of this pathogen followed [6]. The effects of HHV-6 on different cell lines *in vitro* and *in vivo* of neural (oligodendrocytes, astrocytes and neurons), epithelial and fibroblastic origin were reviewed by Braun and colleagues [7]. The process of HHV-6 entry into the host cell is a complex interplay of multiple viral envelope proteins and cellular structures, including the common receptor for nucleated cells CD46 [8]. Persistence in different cells involves both a latent viral life cycle with no production of an infectious virus and a low-level viral replication, each occurring at different anatomic regions [1].

A number of neurological disorders have been associated with the reactivation of herpesviruses during periods of relative immunosuppression reviewed by Yao and colleagues [9]. The role of HHV-6 as a potential trigger of white matter demyelination has generated considerable research interest in the recent years [10,11]. The association between HHV-6 and multiple sclerosis, the most common demyelinating disease of human central nervous system (CNS), was reviewed by Voumvourakis and colleagues [12]. Some other studies suggest that HHV-6 may play a role in the pathogenesis of another demyelinating disorder–progressive multifocal leukoencephalopathy [13]. The high detection rate of HHV-6 genomic sequence in temporal lobe epilepsy patients with a history of encephalitis suggests a potential implication of HHV-6 in the pathological findings of this disorder [14]. Also high cerebrospinal fluid viral loads of HHV-6 have been detected in patients with encephalitis [15]. This may support the possibility of HHV-6 being the cause of the primary encephalitis in patients later developing temporal lobe epilepsy. HHV-6-associated encephalopathy has been reported [16], however, the connection between viral gene expression and disease pathogenesis remains unclear.

The olfactory system exhibits several unique properties that are not typical to other sensory systems. First of all, olfactory receptor neurons directly expose their receptor molecules to the external environment so that they can detect volatile chemical stimuli. Furthermore, these neurons have the capacity for uptake and trans-synaptic transport of exogenous substances to the CNS [17]. It has been shown that airborne pathogens can enter the olfactory pathways and that their transport is usually arrested in the glomerular layer of the olfactory bulb [18]. However, it has also been shown that influenza virus can enter the CNS via the olfactory epithelium [19]. Moreover, Harberts and colleagues have shown that olfactory pathways can be used by HHV-6 to enter the brain and consequently may be implicated in the induction or exacerbation of neurologic disorders [20].

In healthy individuals, glial cells are involved in the maintenance of homeostasis, myelin formation and metabolic support of neurons [21]. In response to brain injury, glial cells can acquire different phenotypes–astrocytes can increase cytokine production and expression of cellular proteins such as S100 [22–24], oligodendrocytes can initiate demyelination [25], and active or phagocytic microglia, the resident immune cell population of the CNS, can promote neuroinflammation [26–28]. Multiple reports have also noted that the olfactory deficit is often accompanied by altered cellular communication and may be one of the first symptoms of neurodegeneration [29–31].

The aim of this study was to analyze structural and ultrastructural alterations in human olfactory pathways and possible associations with HHV-6 infection by 1) detecting HHV-6 genomic sequences, 2) determining the occurrence of the HHV-6 antigen within the olfactory pathways, applying quantitative estimations of HHV-6 immunoexpression in different cell types, and 3) by analyzing cellular and subcellular changes in the case of an unspecified encephalopathy with evidence of HHV-6 infection when compared to healthy age-matched and younger controls.

## Materials and Methods

Brain tissue autopsy samples (olfactory bulb and tract region) from 20 unspecified encephalopathy cases (mean age 63.9 (range 42–78); 12 males and 8 females), 17 age-matched controls (mean age 61.4 (range 49–74); 16 males and 1 female) and 16 young controls (mean age 28.3 (range 18–36); 14 males and 2 females) were used in this study. For investigation of immunohistochemically detected HHV-6 correlations to the PCR positivity, as well as for detection of any differences in tissue structure and ultrastructure between HHV-6 PCR positive and negative cases, individuals in all groups were subdivided as follows: 1) HHV-6 PCR positive (PCR^+^) and HHV-6 PCR negative (PCR^-^) encephalopathy, 2) HHV-6 PCR positive (PCR^+^) and HHV-6 PCR negative (PCR^-^) age-matched controls and 3) HHV-6 PCR positive (PCR^+^) and HHV-6 PCR negative (PCR^-^) young controls.

Autopsy samples were obtained from the Department of Pathology, Riga 1st hospital and the Latvian State Centre for Forensic Medical Examination after a macroscopic and microscopic evaluation made by a certificated pathologist.

The *post mortem* interval was between 7 and 30 hours.

The criteria for inclusion in the encephalopathy group were 1) diagnosis of an encephalopathy determined by pathomorphological examination of the brain, applying measurements and calculations of the cerebral ventricle dimensions relative to the skull as well as by microscopic evidence as described by Chapenko and colleagues [32], 2) no prior history of neurological disorders, cerebrovascular disease, as well as traumatic brain injury, and no history of addiction-inducing substance use 3) common types of encephalopathy (metabolic, toxic, ischemic and hemorrhagic) excluded during the lifetime and/or during autopsy. Controls were created using the same inclusion criteria except the pathomorphological changes consistent with encephalopathy, and younger controls were used to exclude age related changes.

All conventional autopsies were performed and tissue material was investigated in accordance with the laws and requirements of the Republic of Latvia and the European Union (15 December 1992, Law: “On the Protection of the Body of Deceased Human Beings and the Use of Human Tissues and Organs in Medicine”) in the Riga 1st hospital and Latvian State Centre for Forensic Medical Examination. The study protocol and the use of the tissue samples were approved by the Ethics Committee of Riga Stradins University (Decision of the RSU Ethics Committee on 30.05.2013.) and conducted in accordance with the principles stated in the Declaration of Helsinki. All data and information of individuals were anonymized after death and before the conventional autopsies in the Riga 1st hospital and Latvian State Centre for Forensic Medical Examination as well as prior to the authors receiving the tissue samples. Prior to this research project, none of the authors had any relationship with the patients from which the autopsy samples were taken.

### Nested polymerase chain reaction and real-time polymerase chain reaction

Nested polymerase chain reaction (nPCR) and real-time polymerase chain reaction (RT PCR) was used for the qualitative and quantitative detection of viral genomic sequences in DNA isolated from olfactory pathways (both gray and white matter regions) tissue samples.

Total DNA was isolated from tissue samples using standard phenol-chloroform extraction. To ensure the quality of extracted DNA, a β-globin PCR was performed. PCR amplification for the HHV-6 was conducted using 1 μg of the tissues DNA. A method for HHV-6 DNA detection by Secchiero and coworkers was used [33].

HHV-6 load was determined in all HHV-6 positive samples using HHV-6 Real-TM Quant kit (Sacace, Biotechnologies, Italy) and Applied Biosystems 7500 Real-time PCR System (Applied Biosystems, USA) according to the manufacturer's recommendations.

### Immunohistochemistry

HHV-6 (2001) mouse monoclonal antibody raised against viral lysate for immunohistochemical (IHC) detection of A and B strains (Santa Cruz Biotechnology, Inc., Santa Cruz, CA, USA, sc-65463, 1:200) and mouse monoclonal antibody to S100 for detection of intracellular activity of a wide range of cells (Cell Marque, Rocklin, CA, USA, clone 4C4.9, 1:100) were used. IHC reaction with CD68 mouse monoclonal antibody (DacoCytomation, Glostrup, Denmark, clone PG-M1, 1:50) was performed on brain tissue samples to detect activated microglia/macrophages. For quantitative analysis of the presence of CD4 positive and CD8 positive lymphocytes in the autopsy material of the olfactory pathways anti-CD4 (Cell Marque, Rocklin, CA, USA, clone SP35, 1:100) and anti-CD8 (Cell Marque, Rocklin, CA, USA, clone C8/144B, 1:100) mouse monoclonal antibodies were used. IHC for the myelin basic protein (MBP, Santa Cruz Biotechnology, Inc., Santa Cruz, CA, USA, clone 1.B.645, 1:150) and glial fibrillary acidic protein (GFAP, Novocastra, Leica Biosystems, Newcastle, UK, clone GA5, 1:100) was performed. For visualization of antigen-antibody reactions HiDef Detection™ HRP Polymer system (Cell Marque, Rocklin, CA, USA) was used. Standard immunofluorescence staining was performed using goat anti-mouse IgG-FITC: sc-2010 (Santa Cruz Biotechnology, Inc., Santa Cruz, CA, USA, 1:300). For IHC, the antigen sites were visualized with 3,3'-diaminobenzidine (DAB) substrate kit (Cell Marque, Rocklin, CA, USA). The sections were counterstained with Mayer’s hematoxylin and mounted in Roti®-Histokitt (Carl Roth, Karlsruhe, Germany). For immunofluorescence, sections were counterstained with 4',6-diamidino-2-phenylindole (DAPI) (Thermo Fisher Scientific, Invitrogen, UK, 1:3000) and embedded in Prolong Gold with DAPI (Thermo Fisher Scientific, Invitrogen, UK). Tissue autofluorescence was suppressed by 0.2% Sudan Black B solution (Sigma Aldrich, St. Louis, MO) before cover slipping.

Cells that were labeled with the above-mentioned antibodies and displayed brown reaction products were considered immunopositive. Sections from cases with known antibody positivity were used as positive controls. IHC controls that included substitution of the primary antibodies with tris(hydroxymethyl)aminomethane (TRIS) solution were used as negative controls.

IHC analysis included evaluation of immunoreactivity of neurons, glial cells (astrocytes, oligodendrocytes as well as microglia/macrophages) and endothelial cells/vasculature. The total number of immunopositive neurons and glial cells appearing within the vision field reflecting a certain brain region was estimated quantitatively. The expression of antigens was estimated in 10 randomly selected vision fields (for CD4 and CD8 analysis all available tissue material was used resulting in more visual fields examined) of each sample at ×400 maginifcation, using Leica light microscope (LEICA, LEITZ DMRB, Germany) and Glissando Slide Scanner (Objective Imaging Ltd., Cambridge, UK). Reproducible measurements (Aperio ImageScope program v12.2.2.5015) of tissue markers were obtained including gray (olfactory bulb) and white (olfactory tract) matter of the olfactory pathways. For immunofluorescence sections, digital images were captured using Nikon confocal microscope Eclipse Ti-E (Nikon).

### Transmission electron microscopy

1 mm^3^ tissue autopsy material pieces were processed for conventional transmission electron microscopy (TEM) and fixed in 2.5% glutaraldehyde in 0.1 M phosphate, postfixed in 1% osmium tetroxide and embedded in epoxy resin (Sigma-Aldrich, Buchs, Switzerland). Ultrathin, 60-nm thick fine sections were cut with LKB ultramicrotome, collected on Formvar-coated 200-mesh copper grids, and examined in a JEM 1011 transmission electron microscope (JEOL, Japan) at magnification × 8000 to × 25000.

### Data analysis

Apart from descriptive morphology, statistical analysis was performed in order to analyze the IHC results. Data were presented as median with interquartile range (IQR). The Chi-Square test was performed for categorical variables. Comparison between the encephalopathy and control groups was carried out using the Mann-Whitney U test; within the groups–Wilcoxon signed ranks test. A p-value of less than 0.05 was considered statistically significant. The SPSS software 22.0 version was used for all calculations.

## Results

### Nested polymerase chain reaction and real-time polymerase chain reaction

HHV-6 genomic sequence was revealed in 12 out of 20 (60%) tissue samples from the encephalopathy group. HHV-6 load in the 12 nPCR positive samples ranged from <10 to 9715.7 (copies/10^6^ cells). HHV-6 genomic sequence was revealed in 5 out of 17 (29%) tissue samples from the age-matched control group. HHV-6 load in those cases ranged from <10 to 386.5 (copies/10^6^ cells). The presence of HHV-6 genomic sequence was found in 3 out of 16 (19%) tissue samples from the young control group, whereas HHV-6 load <10 (copies/10^6^ cells).

### Immunohistochemistry

#### Differences in immunohistochemical patterns between the encephalopathy cases and controls

A significantly higher number (p < 0.001) of HHV-6 positive neurons was found in both encephalopathy and age-matched control groups when compared to the young controls. In the encephalopathy group both the gray and the white matter had a significantly higher number (p ≤ 0.005) of HHV-6 positive astrocytes, oligodendrocytes, microglial and vascular bed cells when compared to the young control group. Similar differences were found when the young control group was compared to the age-matched control group. In the encephalopathy group a significantly higher number (p ≤ 0.005) of HHV-6 positive astrocytes, oligodendrocytes, microglial and vascular bed cells was found in the white matter whereas in the gray matter only astrocytes (p = 0.012), microglia (p < 0.001) and vasculature (p = 0.006) revealed significant differences when compared to the age-matched control group (Table 1).

**Table 1:** Cellular localization of HHV-6 antigen in the olfactory pathways.

		gray matter			white matter	
	encephalopathy group	age-matched controls	young controls	encephalopathy group	age-matched controls	young controls
	median (IQR)	median (IQR)	median (IQR)	median (IQR)	median (IQR)	median (IQR)
	n	n	n	n	n	n
neurons	3 (1;6)*	3 (2;5)*	2 (1;3)	N/A	N/A	N/A
	157	152	126			
astrocytes	2 (1;4)	2 (1;3)	1 (0;2)	3 (1;5)	2 (0;2)	1 (0;2)
	157	152	126	200	170	153
oligodendrocytes	3 (1;5)*	3 (2;5)*	2 (1;3)	4 (2;7)	3 (2;5)	2 (1;3)
	157	152	126	200	170	153
microglia	2 (1;4)	1 (0;2)	1 (0;2)	3 (1;4)	2 (1;2)	1 (0;2)
	157	152	126	200	170	153
vascular bed	1 (0;2)	1 (0;1)	0 (0;1)	1 (1;2)	1 (0;1)	0 (0;1)
	157	152	126	200	170	153

* The number of HHV-6 positive cells did not differ significantly between the groups.

N/A–does not apply.

**Fig 1:**
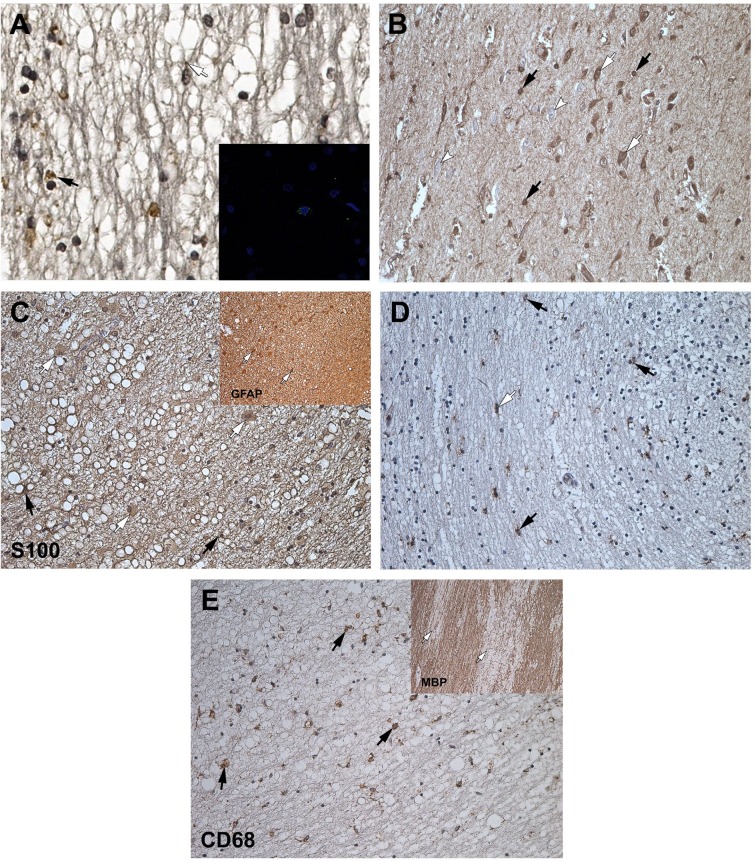
IHC. (A) Immunoreactivity observed in the olfactory tract of HHV-6 positive subjects: HHV-6 immunopositivity in the astrocytes (white arrow) and oligodendrocytes (black arrow), × 250. Confocal microscopy, representative image of HHV-6, insert, × 1000. (B) S100 immunopositivity in the cells of the olfactory bulb: positive neurons (white arrows), negative neurons (arrowheads), astrocytes (black arrows), × 200. (C) S100 and GFAP immunopositivity in the astrocytes (white arrows) and S100 positive oligodendrocytes (black arrows) of the olfactory tract, × 250; insert, × 200. (D) CD68 immunopositivity in the activated microglial cells/macrophages close to the neurons (black arrows) and oligodendrocytes (white arrow) of the olfactory bulb, × 200. (E) CD68 immunopositivity in the activated microglial cells/macrophages (black arrows) and MBP expression (white arrows) of the olfactory tract, × 250; insert, × 100.

**Fig 2:**
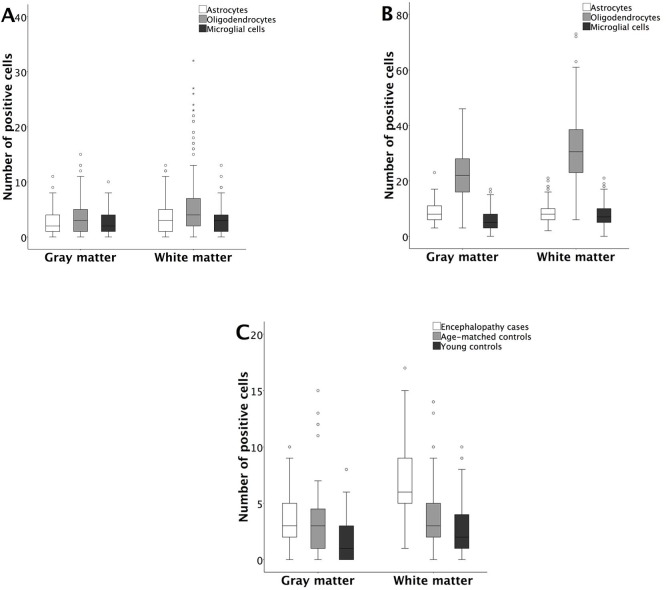
Quantitative comparison of the IHC results between the gray and the white matter. (A) Distribution of HHV-6 positive glial cells in the encephalopathy group. (B) Distribution of S100 positive glial cells in the encephalopathy group. (C) Distribution of CD68 positive activated microglial cells/macrophages in the encephalopathy group and both control groups.

Compared to both control groups, the gray matter of the encephalopathy group revealed significantly more (p = 0.005) HHV-6 positive astrocytes while the white matter revealed significantly more (p < 0.001) astrocytes and oligodendrocytes (Fig 1A and 1A, insert). In the encephalopathy group significantly higher numbers of HHV-6 positive astrocytes (p = 0.004) and oligodendrocytes (p < 0.001) were found in the white matter in comparison to the gray matter (Fig 2A). In the age-matched control group there were significantly more HHV-6 positive oligodendrocytes (p = 0.029) in the white matter as compared to the gray matter. In the young control group significant differences between the cell numbers in the gray and the white matter regions were observed only for astrocytes (p = 0.003).

In the encephalopathy group, there were significantly (p < 0.001) more S100 positive oligodendrocytes and microglial cells in the white matter as compared to the gray matter (Figs 1B, 1C and 2B). Both the gray and the white matter revealed significantly (p < 0.001) higher numbers of S100 positive cells in the encephalopathy group when compared to young controls. Similar results were found when age-matched and young control groups were compared. Encephalopathy group as compared to age-matched controls revealed significantly higher number of S100 positive neurons (p < 0.001), astrocytes (p = 0.010), oligodendrocytes (p = 0.001) and microglial cells (p = 0.004) in the gray matter, while the white matter revealed a significant difference only in the number of microglial cells (p < 0.001) (Table 2).

**Table 2:** Cellular localization of S100 antigen in the olfactory pathways.

	gray matter			white matter		
	encephalopathy group	age-matched controls	young controls	encephalopathy group	age-matched controls	young controls
	median (IQR)	median (IQR)	median (IQR)	median (IQR)	median (IQR)	median (IQR)
	n	n	n	n	n	n
neurons	7 (5;10)	5 (4;8)	3 (2;5)	N/A	N/A	N/A
	159	125	118			
astrocytes	8 (6;11)	9 (7;13)	4 (2;7)	8 (6;10)*	8 (6;14)*	3 (1;10)
	159	125	118	176	160	126
oligodendrocytes	22 (16;28)	15 (11;25)	7 (4;11)	30.5 (23;58.75)*	27 (19;43.75)*	8 (5.75;15)
	159	125	118	176	160	126
microglia	5 (3;8)	4 (3;7)	2 (1;3)	7 (5;10)	5 (3;6)	2 (1;4)
	159	125	118	176	160	126

* The number of S100 positive cells did not differ significantly between the groups.

N/A–does not apply.

Reactive astrocytes with plainly visible cytoplasm were found in the white matter as a common finding within the encephalopathy group (Fig 1C, insert). Although *corpora amylacea* have no clear pathological significance, as compared to both control groups, the white matter of the encephalopathy group revealed significantly more spherical bodies near the astrocytes.

CD68 expression revealed significant differences (p < 0.001) between the encephalopathy group and both control groups (Table 3). In all three groups there were significantly (p < 0.001) more activated microglial cells in the white matter than in the gray matter. There were significantly (p < 0.001) more activated microglial cells in the white matter of the encephalopathy group than in both control groups (Fig 1D and 1E).

**Table 3:** Prevalence of the CD68 positive activated microglial cells/macrophages.

		gray matter			white matter	
	encephalopathy group	age-matched controls	young controls	encephalopathy group	age-matched controls	young controls
	median (IQR)	median (IQR)	median (IQR)	median (IQR)	median (IQR)	median (IQR)
	n	n	n	n	n	n
microglia	3 (2;4.25)	2 (1;4)	1 (0;3)	6 (5;8)	3 (2;5)	2 (1;4)
	166	138	129	200	160	160

The microgliosis in the encephalopathy group was predominantly diffuse, occasionally revealing small microglial clusters. Additionally, multiple foci of white matter demyelination were noted (Fig 1E, insert). We also found similar differences when age-matched and young control groups were compared (Fig 2C).

Results show, that there were more HHV-6 and CD68 positive cells in the encephalopathy group than the controls. The pattern of CD68 positive cells in the encephalopathy group was diffuse with some clustering, whereas in both control groups they were mostly found near capillaries. Significantly more S100 positive oligodendrocytes and activated microglia/macrophages were found in both encephalopathy and age-matched control groups compared to young controls.

#### Differences in immunohistochemical patterns between HHV-6 PCR positive and negative encephalopathy cases and controls

**Fig 3:**
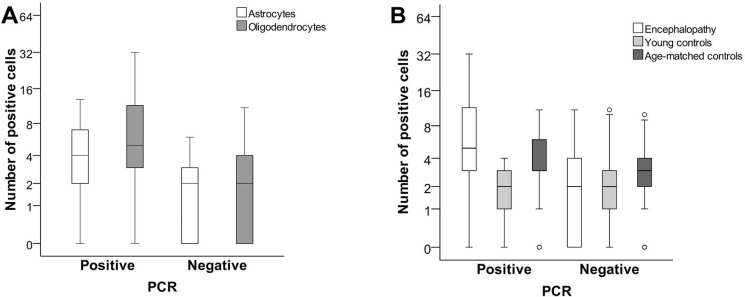
Quantitative comparison of the IHC results between PCR^+^ and PCR^-^ cases. (A) Distribution of HHV-6 positive astrocytes and oligodendrocytes in the white matter of the encephalopathy group. (B) Distribution of HHV-6 positive oligodendrocytes in the white matter of the encephalopathy group compared to controls.

In the PCR^+^ encephalopathy group both the gray and the white matter had a significantly (p ≤ 0.001) higher number of HHV-6 positive astrocytes [3 (2;5) and 4 (2;7), respectively] and oligodendrocytes [4 (2;7) and 5 (3;11.75), respectively], when compared to the PCR^-^ encephalopathy group [2 (0;3) and 2 (0;3); 2 (0;4) and 2 (0;4), respectively] and PCR^-^ age-matched controls [1 (0;2) and 1 (0;2); 0 (2;4) and 3 (2;4), respectively]. Furthermore, there were significantly (p = 0.001) more HHV-6 positive oligodendrocytes [5 (3;11.75)] in the white matter of the PCR^+^ encephalopathy group when compared to both PCR^+^ age-matched [3 (3;6)] and young control groups [2 (1;3)], (Fig 3A and 3B).

No significant differences were found in the number of HHV-6 positive astrocytes or oligodendrocytes in either the gray or the white matter between the PCR positive and negative young control groups, likely attributed to very low HHV-6 positive cell count in both groups.

The number of S100 positive cells was significantly (p≤0.002) lower in the PCR^+^ encephalopathy group when compared to PCR^-^ encephalopathy group–astrocytes in the gray matter 7 (6;9) and 9 (7;12); oligodendrocytes in the white matter 27 (17;37) and 33.5 (27;39), respectively. In contrast, among the age-matched controls the number of S100 positive astrocytes and oligodendrocytes was significantly higher (p≤0.016) in the PCR^+^ group, both in the gray and the white matter. In both, PCR^+^ encephalopathy and control groups, the number of S100 positive oligodendrocytes was significantly higher (p≤0.009) in the white matter.

There were significantly (p < 0.001) more CD68 positive activated microglial cells in the PCR^+^ encephalopathy group when compared to the PCR^-^ encephalopathy group as well as both PCR^+^ control groups. In all PCR^+^ groups, there were more activated microglial cells in the white matter than in the gray matter– 7 (5;9) and 3 (2;5); 6 (4;8) and 5.5 (3;7), 4 (2;5.5) and 1 (0;3) in PCR^+^ encephalopathy, age-matched, and young control groups, respectively.

There was a small number of lymphocytes in the white and gray matter of the encephalopathy group. However, more CD4 positive and CD8 positive cells were found in the white matter than in the gray matter: 1 (0;2) and 0 (0;0.75); 2 (2;4) and 0 (0;3), respectively. We detected a significantly higher number of CD8 positive cells than CD4 positive cells in both gray (p = 0.026) and white matter (p = 0.007) of the encephalopathy group. CD4 positive lymphocytes were predominantly located near the blood capillaries. On the contrary, CD8 positive lymphocytes were located both near the vessels and diffusely in the gray and white matter. HHV-6 PCR^+^ encephalopathy cases showed a significant positive correlation with CD8 lymphocyte count (r = 0.590, p = 0.021).

Overall, the comparisons between only the PCR^+^ encephalopathy and control groups with regard to HHV-6 and CD68 positive cells mirrors the results described in the previous results section. There were more HHV-6 positive oligodendrocytes in the white matter in the PCR^+^ encephalopathy further strengthening the association among the HHV-6 presence and increased number of CD68 positive cells. We found reduced expression of S100 in the glial cells of the PCR^+^ encephalopathy group when compared to the PCR^-^ encephalopathy group. In contrast, PCR^+^ age-matched controls showed increased S100 positivity in glial cells compared to PCR^-^ controls. In spite of these differences, all groups demonstrated significantly higher number of S100 positive cells in the white matter. The number of CD8 positive cells in the white matter was higher compared to the CD4 positive cells while in general the number of lymphocytes was small.

### Transmission electron microscopy

**Fig 4:**
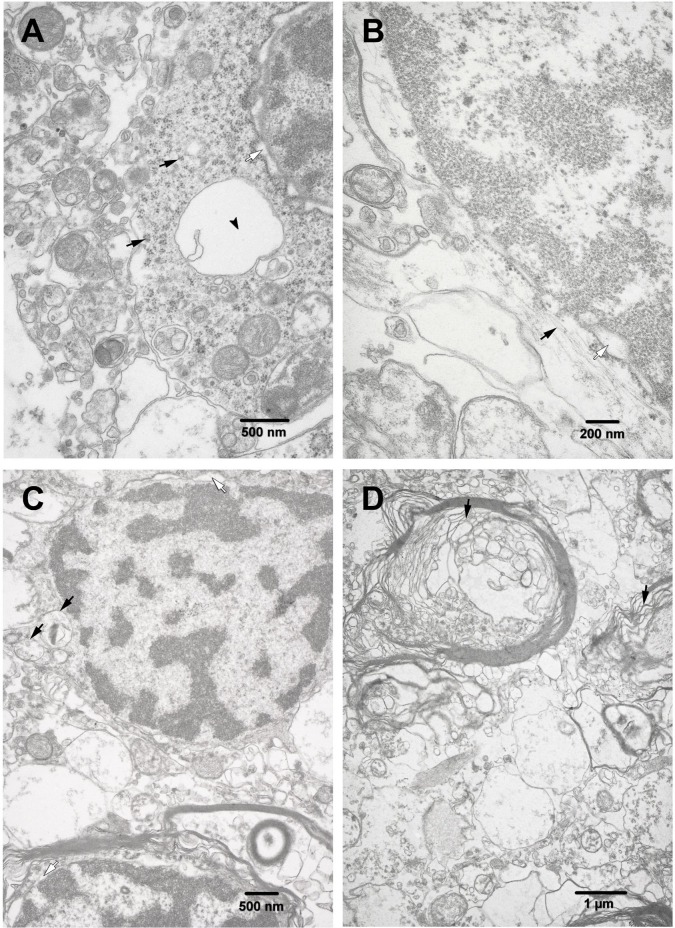
TEM. **Ultrastructural changes observed in the olfactory pathways of HHV-6 positive subjects.** (A) A fragment of neuron revealing irregularly dilated perinuclear space (white arrow), remarkable decrease of rough endoplasmic reticulum, polyribosomes (black arrows), mitochondria and vacuoles (arrowhead). (B) A fragment of astrocyte nucleus, an electron-lucent perinuclear cytoplasm (white arrow) with scanty intermediate filaments (black arrow). (C) Two oligodendrocytes with characteristically structured nuclear chromatin, dilated perinuclear space (white arrow) surrounded by a narrow rim of cytoplasm containing some swollen mitochondria (black arrows), cisternae of rough endoplasmic reticulum, and ribosomes. Myelin layer surrounding axons is thin; profiles of the paranodal loops are greatly varying. (D) Some myelinated axons and dendritic processes. Myelin layer of axons shows irregularity and ballooning of paranodal loops (black arrows).

Ultrastructural examination revealed peculiarities of subcellular damage strongly manifested in the PCR^+^ encephalopathy group as compared to the PCR^-^ group as well as the controls. Neurons often displayed nuclei with a wavy nuclear membrane contour and an expanded perinuclear space, whereas, chromatin mostly showed a fine granular pattern (Fig 4A). Ribosomes and polyribosomes were detected in the perinuclear region. Electron-lucid cytoplasm contained polyribosomes and only occasional profiles of rough endoplasmic reticulum cisternae. Variably shaped mitochondria, lipofuscin inclusions and poorly defined cytoskeleton elements were present in the neuronal perikarya.

Neuropil showed mostly irregularly outlined endings of dendrites and reduced number of dendritic spines in the encephalopathy group. An electron-lucid cytoplasm of dendrites usually contained occasional swollen mitochondria with reduced cristae and expanded cisternae of rough endoplasmic reticulum. Axonal endings showed well-preserved neurofilaments and some microtubules. Synapses found in these areas often lacked vesicles.

Astrocytes presented an electron-lucid cytoplasm with scattered organelles. Elongated and oval shaped nuclei revealed a characteristic chromatin pattern and a well detectable nucleolus (Fig 4B). Cytoplasm showed large, swollen and variably shaped mitochondria, vacuoles and bundles of well-defined intermediate filaments. Foot processes of the astrocytes often appeared to be swollen.

Oligodendrocytes demonstrated a narrowed and electron-dense cytoplasm. It demonstrated profiles of rough endoplasmic reticulum, ribosomes and swollen mitochondria. The nuclei of these cells had mostly round to oval shape, and characteristically distributed clumps of the heterochromatin. Perinuclear space, was irregularly distended (Fig 4C). Sometimes remnants of myelin membranes in the cytoplasm were seen.

The white matter demonstrated numerous and often large-sized filamentous bodies (*corpora amylacea)*. Heterogeneity of the white matter demyelination was confirmed ultrastructurally when examining the olfactory pathways of subjects included in the encephalopathy group. Evidence for severe alterations of the myelin layer of axons was obtained in this group. Myelin sheaths often demonstrated irregularly expanded spaces between the neighboring myelin membranes, crossing and ballooning of lamellae (Fig 4D). Cytoplasmic content of oligodendrocyte entrapped between the myelin membranes was sometimes visible.

Elongated endothelial cells revealed an electron-lucid cytoplasm. Their nuclei often showed a wavy contour of the nuclear membrane. Fewer microvillar projections and pinocytotic vesicles were demonstrated on the surface of endotheliocytes. Cytoplasm contained scanty organelles–occasional swollen mitochondria, profiles of rough endoplasmic reticulum cisternae and vacuoles. Nevertheless, tight junctions between endothelial cells were well-preserved. Thickness of the basement membrane was varying, sometimes exhibiting a multilamellar appearance with the basement membrane layers encircling cytoplasm of pericytes and endothelial cells. Pericytes showed electron-dense cytoplasm containing dilated rough endoplasmic reticulum cisternae, polyribosomes and swollen mitochondria. The nuclear membrane of pericytes was contoured irregularly. Pinocytotic vesicles closely associated with the plasma membrane of pericytes were observed.

Control samples did not demonstrate the above-mentioned ultrastructural changes.

Generally, the main characteristic of the PCR^+^ encephalopathy group found during ultrastructural examination was widespread myelin membrane changes.

## Discussion

The first objective of our study was to look for the presence of the HHV-6 DNA in the olfactory pathways using nPCR and RT PCR methods. Our findings showed higher percentage of HHV-6 positive cases as well as higher viral load values in the encephalopathy group compared to both controls, similarly to Lin and co-authors [34]. Subsequently, we compared the number of HHV-6 positive neural cells in the gray and the white matter of the olfactory pathways as well as tried to discern which of the neural cell populations are more susceptible to viral invasion using the IHC method.

It is known that several viruses can gain entrance into the host cell via direct fusion of the viral envelope with the target-cell plasma membrane or endocytosis followed by fusion of viral and cellular membranes in the endosomal compartment, besides it requires specific receptors [8]. After entering the cell, virus can either persist intracellularly in a latent state or reactivate [1]. In the review article by Reynaud and Horvat in 2013, data from several research papers showing the evidence of viral particle presence in neurons, oligodendrocytes and astrocytes were summarized, also proving that all of these cell types possess the receptors necessary for the fusion process and viral entry into the cell [35]. Our IHC results are in accordance with these findings. In our study all cell types in both the gray and the white matter of the encephalopathy group revealed HHV-6 positivity. Furthermore, significantly higher HHV-6 positivity was demonstrated in astrocytes and oligodendrocytes, although HHV-6 proteins were also present in neurons, microglia and vasculature. Similarly to Lin and colleagues, we also found HHV-6 positive cells in the age-matched controls [34], but the number of such cells was considerably larger in the encephalopathy group. Some HHV-6 positive cells were also found in the young control group.

Our further observations revealed significant differences in HHV-6 positivity between the gray and the white matter. The white matter of the encephalopathy group as well as the controls demonstrated a higher number of HHV-6 positive glial cells than the gray matter. These findings are similar to those of Challoner and colleagues, and data on the white matter lesions by Moritani and colleagues [36,37]. Analyzing regions of the white matter, where we found the largest number of HHV-6 positive cells in both study and control groups, the greatest number of the HHV-6 positive cells was detected among the oligodendrocytes. Moreover, in the case of an encephalopathy, the number of positive oligodendrocytes was significantly higher than that in the control groups. Even though we also found a considerable number of HHV-6 positive astrocytes and their HHV-6 positivity correlated strongly with the HHV-6 positivity of the vasculature, the involvement of oligodendrocytes in HHV-6 mediated changes seems more likely.

The discussion so far has debated the results obtained using the IHC method to analyze the 3 study groups independently from PCR results. However, PCR is recognized as the “gold standard method” for virus DNA sequence detection demonstrating a minor rate of false negative cases [14]. On the other hand, IHC does not exclude false positive results (e.g., by staining *corpora amylacea*). Therefore, an additional IHC analysis was performed dividing each study group in PCR^+^ and PCR^-^ subgroups. Results showed that in both, the PCR^+^ encephalopathy and in the PCR^+^ age-matched control groups, the number of HHV-6 positive cells was significantly higher than in the PCR^-^ encephalopathy and control groups. This indicates that a strong correlation exists between the presence of positive cells labeled by the IHC and the presence of HHV-6 DNA sequences detected by the PCR technique, even though some immunohistochemically HHV-6 positive cells were also detected in PCR^-^ samples, possibly due to partial unspecificity of the antibodies used.

Alterations in the ultrastructure of oligodendrocytes, the cells responsible for production and maintenance of the myelin layer, were also demonstrated using the electron microscopy method, which revealed pathological changes in the cytoplasm and the myelin sheath of axons in the case of PCR^+^ encephalopathy.

The fact that we found more HHV-6 positive cells in the white rather than the gray matter of the olfactory pathways possibly indicates that, if the route of entry of the virus into the CNS is the olfactory epithelium [20], then there should be some sort of favorable microenvironmental factors that promote viral transport from the gray matter of the olfactory bulb to the white matter of the olfactory tract. Furthermore, there are studies introducing the role of the glomerular layer of the olfactory bulb in arresting the movement of the pathogens beyond it [18], but we did not find any evidence of the presence of the virus within the olfactory glomeruli.

Numerous *corpora amylacea* in the white matter of the encephalopathy and age-matched controls were an interesting finding of the current study. In line with the conclusions of Blumberg and colleagues, we did not analyze *corpora amylacea* which stained brown-positive when the IHC method for identifying HHV-6 was used [38].

The next objective of our study was to investigate cellular reactions under the conditions of HHV-6 positivity. It is known that the Ca^2+^-binding S100 protein may exert trophic or toxic effects on neurons and glial cells depending on its concentration [23]. Our results showed decreased expression of S100 by astrocytes in the gray matter and oligodendrocytes in the white matter of the PCR^+^ encephalopathy group when compared to the PCR^-^ encephalopathy group. In addition, we also observed significant differences between the study groups when correlations between HHV-6 positive and S100 positive cells were analyzed–in the encephalopathy group we found only negative correlations between S100 positive and HHV-6 positive glial cells in both the gray and the white matter while control groups revealed positive or no correlations (data not shown). According to Zhou and colleagues, viruses can alter Ca^2+^ homeostasis and utilize Ca^2+^ as well as cellular Ca^2+^-binding proteins to create a cellular environment suitable for their replication, gene expression, maturation and other needs [39]. The reduced expression of S100 in the glial cells of the PCR^+^ encephalopathy group when compared to the PCR^-^ group might represent depletion of these intracellular resources due to long standing infection.

We found a higher number of activated microglial cells in the white matter than in the gray matter, leading us to believe that the white matter is more actively involved in the pathological process in the case of an unspecified encephalopathy. These results were similar to those demonstrated by Olah and colleagues [40]. The results also showed that there were significantly more activated microglial cells in the white matter of the PCR^+^ encephalopathy group than the PCR^-^ group, possibly suggesting a virus induced response. Activated microglia appears to be involved both in the inhibition of viral replication and in neurotoxicity [41]. In the gray matter the highest activity of microglial cells was observed near the neuronal cell bodies. The changes evident in the gray matter were also present in the ultrastructural level showing neurons of PCR^+^ encephalopathy group cases with an altered nuclear and membrane structure, as well as considerable changes of the rough endoplasmic reticulum. These alterations together with up-regulation of the S100 expression could indicate an active process of Ca^2+^ mobilization from internal sources in response to virus infection or expression of viral proteins [39]. At the same time the ultrastructural analysis of perikarya showed a high number of both free and polyribosomes evidencing active cellular synthesis of proteins.

The IHC correlations between the HHV-6 positive glial cells and vasculature, and further ultrastructural examination of the blood-brain barrier components in the case of PCR^+^ encephalopathy, revealed alterations in the structure of endothelial cells, pericytes and the basement membrane. These findings suggest that HHV-6 could be involved in the structural changes of the blood-brain barrier.

The involvement of cellular immune responses in neurodegenerative disorders has been reported [42]. Indeed, our finding of the lymphocytes present within the olfactory pathways may indicate that the immune system attempts to control the HHV-6 infection, possibly leading to inflammation and eventual demyelination. More cytotoxic (CD8 positive) than helper (CD4 positive) lymphocytes were found in the white matter of the encephalopathy group. However, the number of CD8 lymphocytes was not high, in fact, not all of the IHC and nPCR positive cases even had lymphocytes in the white matter of the olfactory tracts.

The HHV-6 also has been shown to persist in the different anatomic sites including salivary glands, and viral DNA can be routinely detected in saliva using PCR [43]. It is possible that the virus may enter nerve endings at the peripheral region and then travel further to the perikarya [44] possibly making contact with the glial cells in its way. The results of a study by Roussarie and colleagues show that the neurotropic Theiler's murine encephalomyelitis virus primarily infects neurons, and the optic nerve oligodendrocytes are infected later on through the myelin sheaths even when the axons do not degenerate [45]. The virus later persists in the oligodendrocytes and macrophages of the white matter. Our results may indicate a similar process to that described by Roussarie, for, although we detected the HHV-6 positivity in the microglial cells, the macrophages of the CNS, other types of glial cells, especially the oligodendrocytes, showed a greater HHV-6 positivity. Our results seem to be in accordance with the conclusions made by Australian researchers stating that the HHV-6 virus does not exert its pathological effects directly via reactivation, but more likely by inducing a pathological process within the CNS cells earlier in the host’s life [46].

From comparison of the whole encephalopathy group to controls as well as PCR^+^ to PCR^-^ encephalopathy cases and controls, we can conclude that differences are especially pronounced in the white matter. An increased number and a stronger association between the HHV-6 positive glial cells and activated microglia/macrophages in the PCR^+^ encephalopathy group compared to PCR^-^ encephalopathy cases and both PCR positive and negative controls indicate an ongoing neuroinflammation process when virus along with the signs of already progressed encephalopathy is present. Furthermore, a significant downregulation of S100 expression in HHV-6 PCR^+^ encephalopathy cases was found as another indicator of possible long-standing inflammatory process. The aforementioned changes point to vulnerability of the white matter thus contributing to the demyelination, also evidenced ultrastructurally.
